# Mechanical shear flow regulates the malignancy of colorectal cancer cells

**DOI:** 10.1002/kjm2.12844

**Published:** 2024-05-17

**Authors:** Yu‐Ting Tseng, Ching‐Chung Tsai, Ping‐Chen Chen, Bo‐Yan Lin, Sodio C. N. Hsu, Shu‐Ping Huang, Bin Huang

**Affiliations:** ^1^ Department of Biomedical Science and Environmental Biology, College of Life Science Kaohsiung Medical University Kaohsiung Taiwan; ^2^ School of Medicine, College of Medicine I‐Shou University Kaohsiung Taiwan; ^3^ Department of Pediatrics, E‐Da Hospital I‐Shou University Kaohsiung Taiwan; ^4^ Department of Biological Sciences National Sun Yat‐sen University Kaohsiung Taiwan; ^5^ Department of Medicinal and Applied Chemistry Kaohsiung Medical University Kaohsiung Taiwan; ^6^ Regenerative Medicine and Cell Therapy Research Center Kaohsiung Medical University Kaohsiung Taiwan; ^7^ Department of Medical Research Kaohsiung Medical University Hospital Kaohsiung Taiwan

**Keywords:** apoptosis, colorectal cancer, EMT, mitochondria, shear flow

## Abstract

Colorectal cancer (CRC) is notable for its high mortality and high metastatic characteristics. The shear force generated by bloodstream provides mechanical signals regulating multiple responses of cells, including metastatic cancer cells, dispersing in blood vessels. We, therefore, studied the effect of shear flow on circulating CRC cells in the present study. The CRC cell line SW620 was subjected to shear flow of 12.5 dynes/cm^2^ for 1 and 2 h separately. Resulting elevated caspase‐9 and ‐3 indicated that shear flow initiated the apoptosis of SW620. Enlarged cell size associated with a higher level of cyclin D1 was coincident with the flow cytometric results indicating that the cell cycle was arrested at the G_1_ phase. An elevated phosphor‐eNOS^S1177^ increased the production of nitric oxide and led to reactive oxygen species‐mediated oxidative stress. Shear flow also regulated epithelial–mesenchymal transition (EMT) by increasing E‐cadherin and ZO‐1 while decreasing Snail and Twist1. The migration and invasion of sheared SW620 were also substantially decreased. Further investigations showed that mitochondrial membrane potential was significantly decreased, whereas mitochondrial mass and ATP production were not changed. In addition to the shear flow of 12.5 dynes/cm^2^, the expressions of EMT were compared at lower (6.25 dynes/cm^2^) and at higher (25 dynes/cm^2^) shear flow. The results showed that lower shear flow increased mesenchymal characteristics and higher shear flow increased epithelial characteristics. Shear flow reduces the malignancy of CRC in their metastatic dispersal that opens up new ways to improve cancer therapies by applying a mechanical shear flow device.

## 1 INTRODUCTION

Colorectal cancer (CRC) is characterized by its poor diagnosis, high metastases, and mortality.^1^ In recent decades, several newly developed strategies, including targeted therapy and CAR‐T cell‐based immunotherapy, were developed.^2^, ^3^ Recent efforts also suggested that mechanical stimuli‐driven cancer therapy can provide a direct therapeutic effect or a mediator to augment traditional cancer therapy.^4^, ^5^ In general, shear flow is essential for maintaining physiologies and lifespan of endothelial cells (ECs), the regulatory effects of shear flow on circulating tumor cells (CTCs) were also reported.^6^, ^7^ The metastatic CTCs are commonly stimulated by shear force, compression, and tensile strain derived by the bloodstream.^8^ High shear stress promotes the production of reactive oxygen species (ROS) and induces apoptosis in lung cancer cells.^9^ The viability of metastatic ovarian cancer cells was significantly impaired by shear stress.^10^ Under an undulating shear force generated by a specific device, the circulating breast cancer cells showed a significant injury while remaining without noticeable effects on human blood cells, demonstrating that CTCs are more susceptible to shear stress.^11^ Controversially, breast cancer cells exposed to shear flow (20 dynes/cm^2^) showed a higher survival rate and an enhanced epithelial–mesenchymal transition (EMT) indicating the promotive effect of shear flow on cancer metastasis.^12^ In lung cancer, shear stress induced the ratio of side populations and finally reduced the survival of rodents with severe metastasis.^13^ As for colon cancer, in circulating human colon cancer cells HCT116, high shear stress (60.5 dyne/cm^2^) impaired cell viability, whereas it promoted cell proliferation by enhancing the levels of β‐catenin and c‐myc.^14^

The mechanosensors such as mechanosensitive ion channels (MIChs) Piezo and TRP families, integrin, lamin A/C, and PAR‐1 involved in cancer physiological responses were identified.^15^ Regulating the functions of mechanical sensors and the downstream components to ameliorate cancer metastasis and malignancy were proposed in diverse cancers.^16^, ^17^ In general, the larger the cancer cell, the lower its energy metabolism and occurrence of cancer.^18^ Therefore, we applied measurements of cell size and cell cycle status to evaluate the effects of shear flow on CTC. ROS and reactive nitrogen species (RNS) are the two free radicals revealing a significant toxicity to cancer cells.^19^ Nitric oxide (NO) and ROS lead to the massive production of additional toxic RNS, such as peroxynitrate (ONOO^−^) that further enhance the therapeutic effect.^20^

Mitochondria are key regulators in energy and metabolite homeostasis. Targeting mitochondria for cancer therapy was widely reported.^21^ CRC cells utilize autophagy to maintain mitochondrial metabolism for cell proliferation under nutrient stress indicating the essential function of mitochondria in cancer survival.^22^ In gastric cancer, transplanting heterologous mitochondria purified from human umbilical vein ECs altered the function of endogenous mitochondria and promoted, therefore, tumor growth.^23^ Under shear flow, the induced signaling cascades implicated in anti‐oxidation, mitochondrial biogenesis, mitophagy, and metabolic homeostasis were widely discussed.^24^, ^25^ Currently, the effect of shear flow on the mitochondrial function of cancer cells was only reported for breast cancer by Park et al.^12^ How shear flow affects the mitochondrial function and leads to the altered characteristics in CRC cells was proposed herein.

The reported article showed a novel device that can create a variable flow speed for eliminating the CTC without damaging blood cells.^26^ On average, the flow speed of the carotid artery is 11.6 dynes/cm^2^, the coronary artery ranges from 5.7 to 43.9 dynes/cm^2^. In the majority of studies, shear flow strengths of 12–25 dynes/cm^2^ were mainly applied.^24^ In a preliminary study, the CRC cells SW480, SW620 and HCT116 were exposed to 12.5 dynes/cm^2^, where only SW620 showed a significant apoptosis. SW620 also represents a human colorectal adenocarcinoma cancer cell with the highest metastatic characteristics that is widely used as a model for colorectal carcinoma and tumor progression.^27^ Dispersing metastatic cells that constantly circulate in the blood vessels are commonly impacted by shear flow. Therefore, SW620 was used in our studies for their changes in cell size, cell cycle, oxidative stress, EMT expression, and mitochondrial function. The expression of EMTs between different flow speeds, 6.25 and 25 dynes/cm^2^, were additionally compared. Therefore, the possible application of mechanical shear flow in eliminating the malignancy of CRC was investigated in the present study.

## 2 MATERIALS AND METHODS

### 2.1 Cell culture

The human colon carcinoma cell line SW620 was purchased from Thermo Fisher Scientific (Rockford, IL, USA). Cells were cultured at high glucose (4.5 g/L) Dulbecco's modified Eagle's medium (DMEM) supplemented with fetal bovine serum (FBS; 10%, v/v), streptomycin (100 μg/mL), and penicillin (100 U/mL). Cells were incubated overnight in a starvation medium (DMEM containing 2% FBS) in a 37°C growth chamber with CO_2_ (5%, v/v) before shear flow treatment.

### 2.2 Shear flow treatment

The SW620 cells were suspended in a DMEM medium containing 2% (v/v) FBS and added into an aseptic silica tube with a 3 mm inner diameter. The shear flow was triggered by of rotary pump set at 0.2, 0.4, and 0.8 mL/sec to generate 6.25, 12.5, and 25 dynes/cm^2^ shear force following a previously published protocol.^24^

### 2.3 Cell apoptosis and necrosis

The SW620 cells (1 × 10^5^), either with static treatment or exposed to different shear flow treatments for 1 and 2 h, were collected by centrifugation (3000 rpm, 5 min). The re‐suspended cells were co‐incubated with 10 μM 7‐AAD and 0.25 μg/mL Annexin V for 30 min and then analyzed by flow cytometry (Guava easyCyte, Luminex Comp. Austin, TX, USA). A background fluorescence of 7‐AAD (*λ*ex 546 nm/*λ*em 647 nm) or Annexin V (*λ*ex 490 nm/*λ*em 525 nm) was obtained from cells separately stained by 7‐AAD or Annexin V.

### 2.4 Western blotting

The total protein content of SW620 cells treated with different shear flow and duration time were extracted by lysis buffer: EDTA (1 mM), HEPES (50 mM, pH 7.7), neocuproine (0.1 mM), and CHAPS (0.4% w/v). About 40 μg of proteins was mixed with sample buffer: Tris–HCl (62.5 mM, pH 6.8), 2‐mercaptoethanol (5%, v/v), SDS (3%, w/v), and glycerol (10%, v/v), and then separated by SDS‐PAGE. The gel was blotted on a PVDF membrane (Millipore, Billerica, MA, USA) and then incubated with antibodies. The antibodies caspase‐3 (1:1000), cleaved caspase‐9 (1:1000), cyclin B1 (1:2000), cyclin D1 (1:2000), eNOS (1:2000), peNOS^S1177^ (1:2000), E‐cadherin (1:2000), and Actin (1:3000) were purchased from Cell Signaling Technology (Danvers, MA, USA). Snail (1:1000), ZO‐1 (1:1000), and Twist 1 (1:1000) were purchased from BD (San Jose, CA, USA). The membranes were developed with the SuperSignal West Femto Maximum Sensitivity Substrate (Thermo Fisher Scientific) and observed by a luminescence imaging device (MiniChemi 500, Sagecreation Co, Ltd, Beijing, China). The protein level was calculated by the Progenesis Samespots v2.0 software (NonLinear Dynamics, Newcastle, UK).

### 2.5 Measurement of cell size

The sheared SW620 cells were collected by centrifugation (3000 rpm, 5 min) and were re‐suspended in 1 mL starvation medium. 1 × 10^5^ cells were analyzed by Scepter™ 3.0 handheld cell counter (Merck, Taufkirchen, Germany) for quantifying the distribution of cell sizes.

### 2.6 Detection of oxidative stress

For the detection of shear flow‐induced NO, the sheared SW620 cells were incubated with 10 μM FA‐OMe (5‐amino‐2‐(6‐hydroxy‐3‐oxo‐3H‐xanthen‐9‐yl)) benzoic acid methyl ester fluorescent probe for 1 h following a previous study.^28^ The average level of NO was calculated from 5 × 10^4^ cells by flow cytometry at *λ*ex 460 nm/*λ*em 524 nm. The level of ROS was measured by DCFDA (2′,7′‐dichlorofluorescin diacetate) kit (Abcam, Waltham, MA, USA). The 5 × 10^4^ SW620 cells were incubated with 5% (v/v) DCFDA for 30 min and then analyzed by flow cytometry at *λ*ex 485 nm/*λ*em 535 nm.

### 2.7 Wound healing assay

The sheared SW620 cells were cultured in a culture plate for 24 h. The gap between cells on the plate was created by a scratch with a 200 μL tip. The images from an initial scratch and after the recovery culture for 24 h were captured by a digital camera (SONY DSC‐H20, SONY Corp. Minato‐ku, Tokyo, Japan) and then analyzed by TScratch software.

### 2.8 Cell invasion assay

Corning™ Matrigel™ (50 μL, Thermo Fisher Scientific) was added into the transwell (pore size 8 μm, Greiner Bio‐One Inc.) and incubated at 37°C for 2 h. Sheared SW620 (5 × 10^4^ cells) were suspended in 25 μL DMEM medium (FBS‐free) and then seeded on the surface of the Matrigel. The transwell was assembled with the 24‐well plate containing DMEM medium (10% FBS) and moved to a 37°C incubator for 24 h. The cells were fixed by paraformaldehyde (4%, w/v) for 15 min and then stained by Giemsa stain (10%, v/v, Sigma‐Aldrich, St. Louis, MO, USA) for 15 min. The redundant dye was removed by washing with H_2_O and then observed by microscopy.

### 2.9 Measurement of mitochondrial mass

After shear flow treatment, a sample of 1 × 10^6^ SW620 cells was collected by centrifugation. The cells were lysed with 500 μL of cytosol extraction buffer by moderate shaking for 20 min. After centrifugation (1000 rpm, 20 min), the supernatant was transferred to a new tube for additional centrifugation (10,000 rpm, 20 min) to precipitate the mitochondria according to the user's guideline (Abcam). The putridity of isolated mitochondria was identified by the expression of COX IV protein in the western blot analysis. The mitochondria harvested from cells were lysed by sonication with 100 μL of lysis buffer (250 mM HEPES pH 7.7, 1 mM EDTA, 0.1 mM neocuproine, and 0.4% (w/v) CHAPS). After centrifugation, the supernatant was transferred to a new tube and the concentration of mitochondrial protein (mt‐protein), also indicated as protein mass, was determined by a BCA reagent (Thermo Fisher Scientific).

### 2.10 Evaluation of mitochondrial membrane potential

The sheared SW620 cells were collected by centrifugation and washed once with phosphate‐buffered saline (PBS) buffer. Cells were incubated with JC‐1 reagent for 30 min according to the user's guideline (Thermo Fisher Scientific). The cells were also incubated with 200 μM mitochondrial membrane potential (MMP) inhibitor carbonyl cyanide m‐chlorophenyl hydrazone (CCCP) for 30 min as a negative control. Fluorescence was measured by flow cytometry from 1 × 10^5^ cells with a combination of red light (*λ*ex 488 nm/*λ*em 661 nm) and green light (*λ*ex 488 nm/*λ*em 525 nm).

### 2.11 Measurement of ATP level

According to the user's guidelines for ATP assay kit (Abcam). In brief, we collected 2 × 10^5^ cells that were re‐suspended in 1 mL PBS buffer and lysed by 250 μL lysis buffer. Aliquoted 50 μL of cell lysate was incubated with 50 μL of assay buffer for 30 min. The ATP level was determined by an ELISA plate reader (Multiskan EX, Thermo Fisher Scientific) at OD 480 nm.

### 2.12 Statistical analysis

Each treatment was repeated for a minimum of three times. Data were expressed as means with standard error of the mean (SEM). The SW620 cells of static treatment were used as a standard to calculate the relative fold of different shear flow conditions. Same letter indicates no significant difference according to Fisher's least significant difference (LSD) post hoc test. Values of *p* < 0.01 were considered as significant.

## 3 RESULTS

### 3.1 Shear flow‐induced cell apoptosis

**FIGURE 1:**
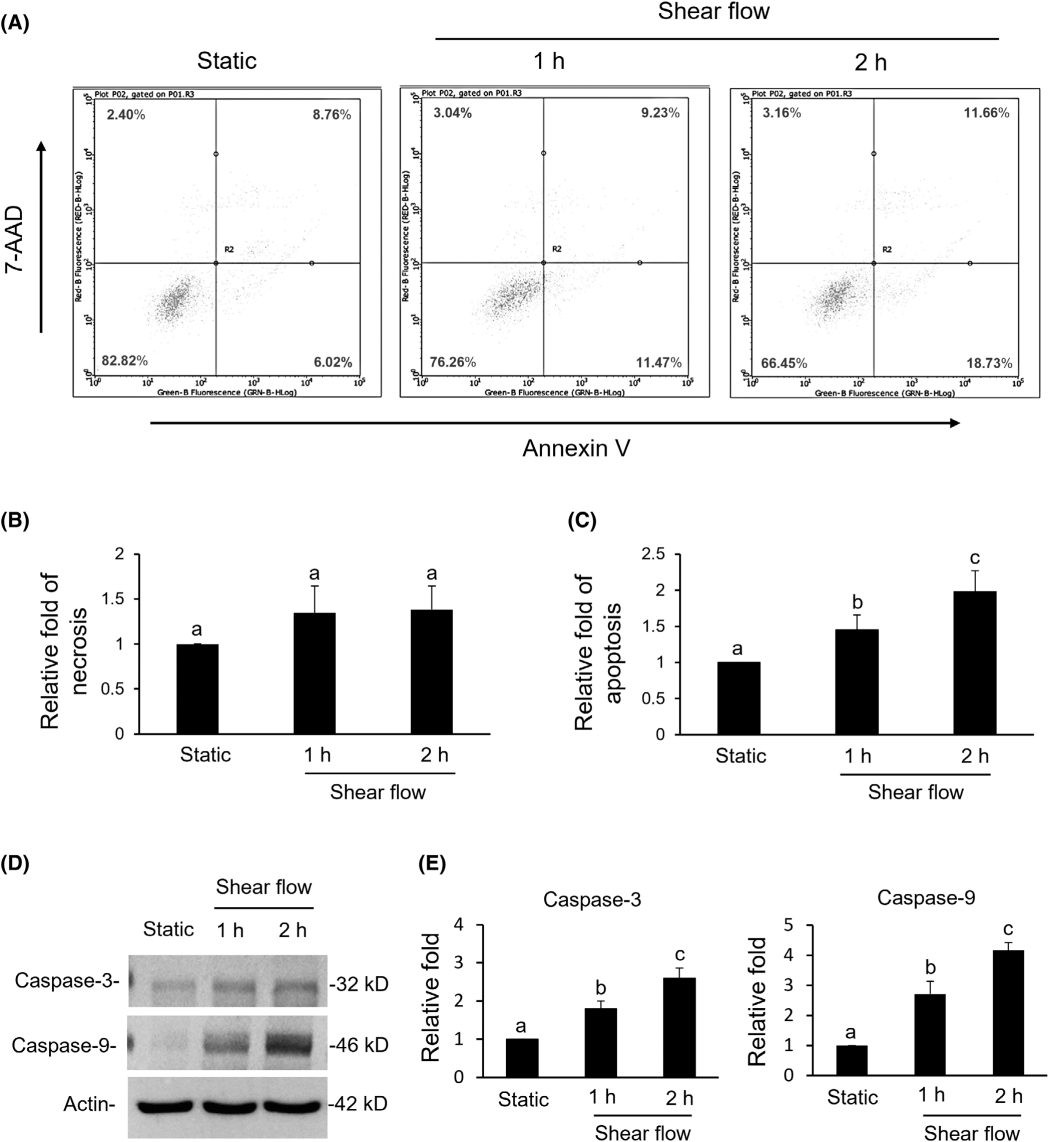
Cell viability assay. (A–C) SW620 cells (1 × 10^5^), either from static or shear flow treatments for 1 and 2 h, were double stained by 7‐AAD and Annexin V for 30 min and then analyzed by flow cytometry. (D and E) Expressions of caspase‐3 and the cleaved caspase‐9 were identified by western blotting. A static treatment was used as a reference to show the relative fold changes of each treatment. Data were provided as means ± SEM. Same letter indicates no significant differences according to Fisher's least significant difference (LSD) post hoc test. Values of *p* < 0.01 were considered as significant.

By applying 7‐AAD and Annexin V, the sheared SW620 cells showed significant apoptosis but no necrosis in the first hours of shear flow and got dominant as shear flow was extended to 2 h (Figure 1A–C). As for apoptotic proteins, caspase‐3 and ‐9 were induced in the first hour of shear flow and elevated in the second hour (Figure 1D,E).

### 3.2 Shear flow increased cell size and arrested cell cycle

**FIGURE 2:**
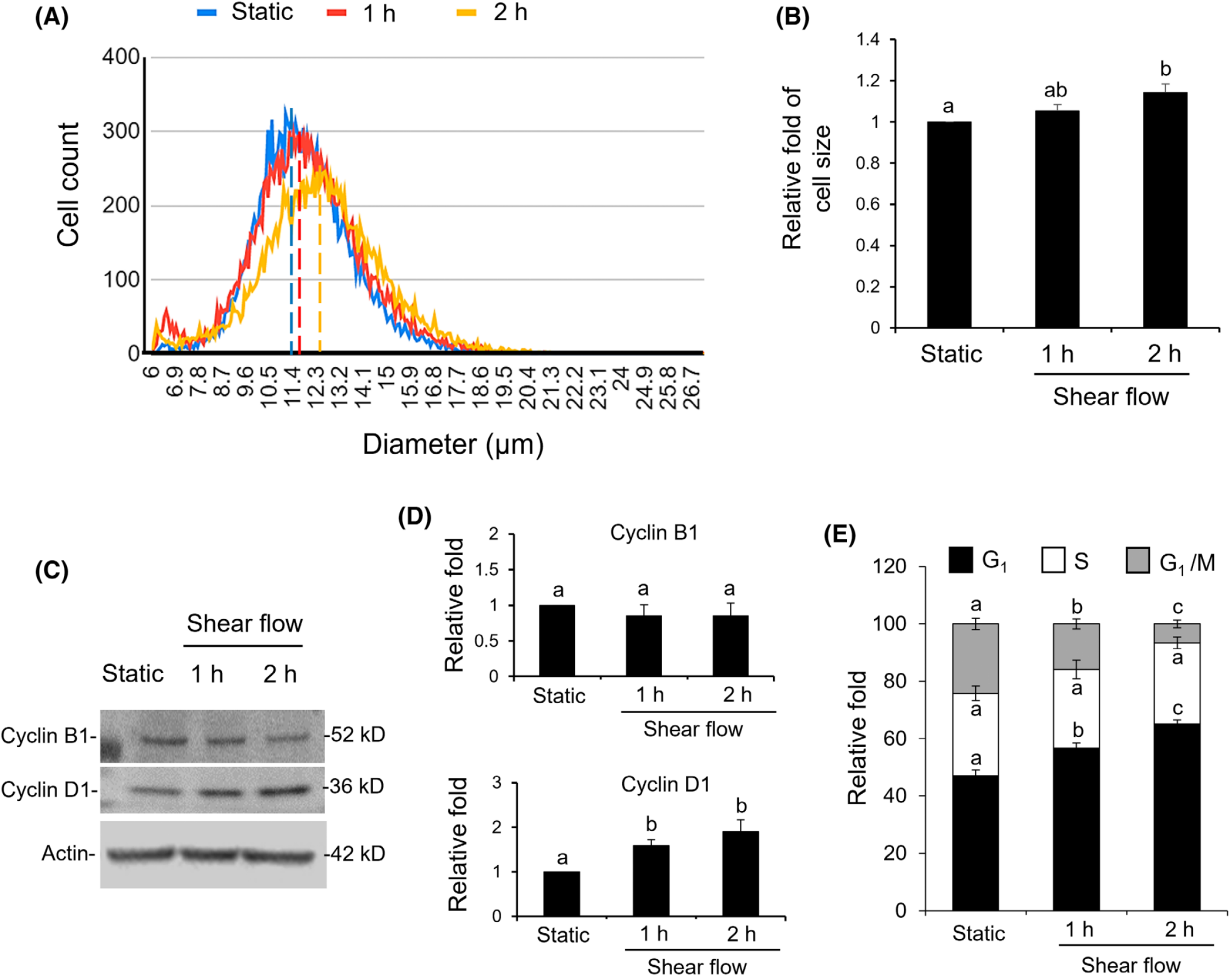
Measurement of cell size and cell cycle after shear flow. (A and B) Cell diameters of 1 × 10^5^ cells from static (blue), shear flow for 1 h (red) and 2 h (yellow) were separately measured by a handheld cell counter. (C and D) Expressions of cyclin B1 and D1 proteins were detected by western blotting. (E) Levels of different cell cycle phases, G1, S, and G1/M from static and sheared cells were compared. A static treatment was used as a reference to show the relative fold changes of each treatment. Data were provided as means ± SEM. Same letter indicates no significant differences according to Fisher's least significant difference (LSD) post hoc test. Values of *p* < 0.01 were considered as significant.

For the 1 × 10^5^ cells that were sheared for 1 h, the cell size remained constant (around 11.4 μm). As shear flow was extended to 2 h, the cell diameter was significantly enlarged compared to a static treatment (11.4 μm) to 12.3 μm (Figure 2A,B). Further analysis of protein expressions involved in the cell cycle showed that the cyclin B1 level was not changed whereas cyclin D1 significantly increased. This indicated that the cell cycle was arrested at the G1 phase (Figure 2C–E).

### 3.3 Shear flow increased oxidative stress

**FIGURE 3:**
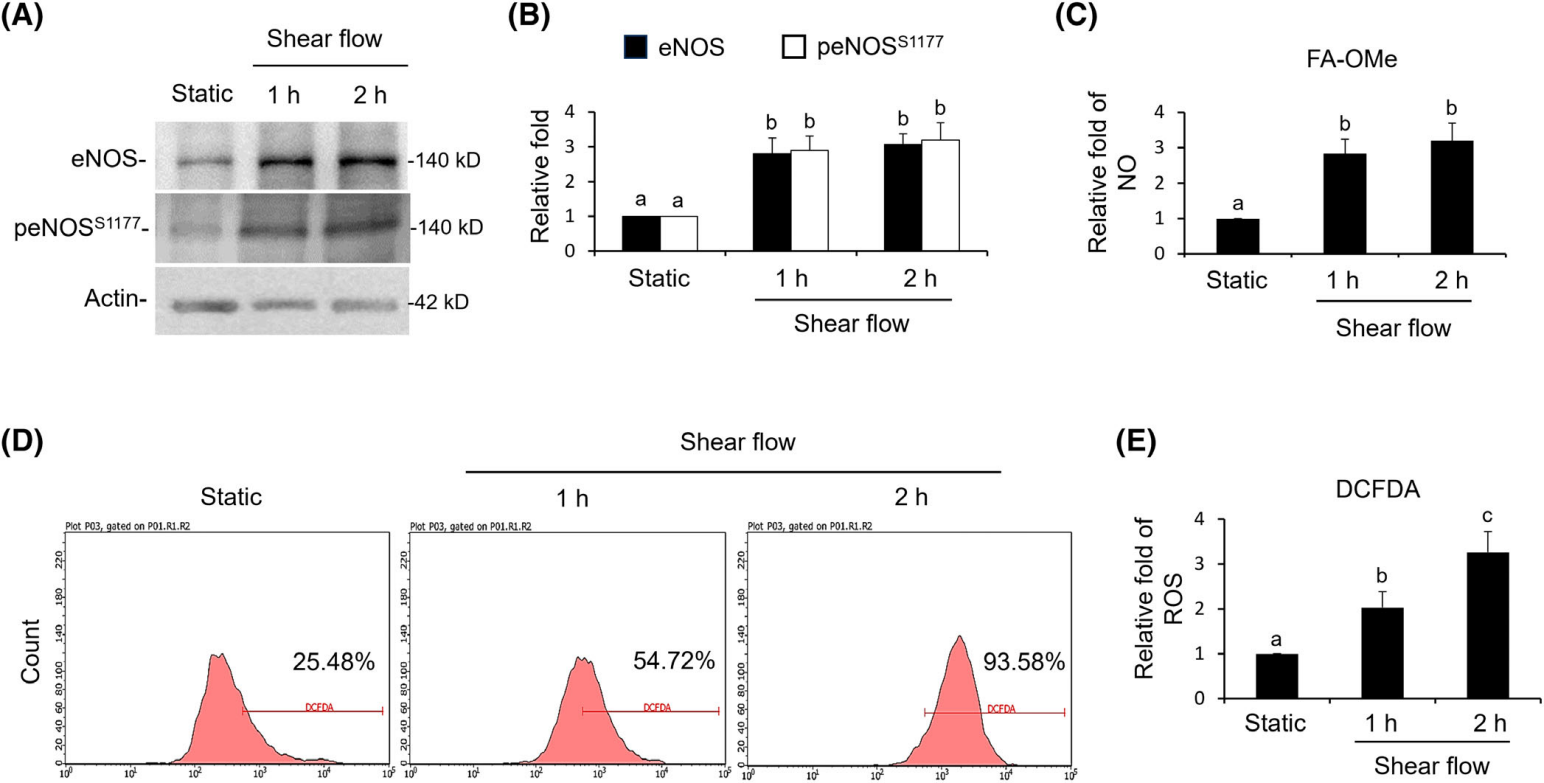
Shear flow increased the production of NO and ROS. (A and B) Expressions of eNOS and peNOS^ser1177^ involved in NO production were identified by western blot. (C) Productions of gaseous NO from 5 × 10^4^ cells were detected by specific fluorescent reagent FA‐OMe. (D and E) Cytosolic ROS of 5 × 10^4^ cells were detected by DCFDA reagent. A static treatment was used as a reference to show the relative fold changes of each treatment. Data were provided as means ± SEM. Same letter indicates no significant differences according to Fisher's least significant difference (LSD) post hoc test. Values of *p* < 0.01 were considered as significant. NO, nitric oxide; ROS, reactive oxygen species.

Oxidative stress was evaluated via the production of NO and ROS. Under shear flow for 1 h, the protein expressions of eNOS and phosphor‐eNOS^s1177^ were significantly elevated. The fluorescent strength of gaseous NO that was detected by the specific reagent FA‐OMe was also threefold higher as compared to the static treatment (Figure 3A–C). As for cytosolic ROS detected by DCFDA reagent, shear flow significantly increased the production of ROS from a 1 h to a 2 h treatment (Figure 3D,E).

### 3.4 The effect of shear flow on the malignancy of SW620 cells

**FIGURE 4:**
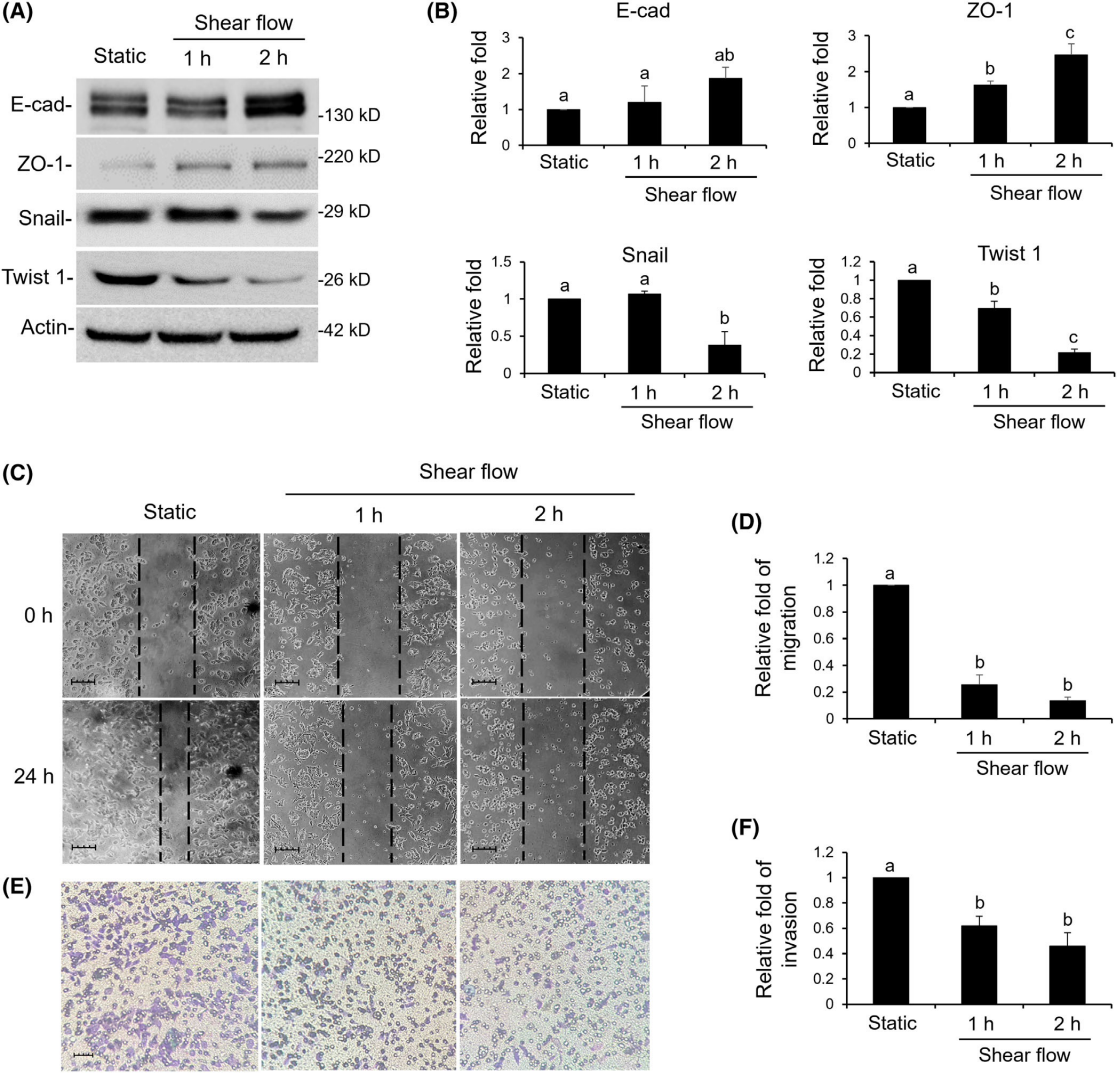
Shear flow altered the expression of EMT proteins, cell migration, and invasion ability. (A and B) Expressions of E‐cad, ZO‐1, Snail, and Twist 1 involved in EMT were identified by western blotting. (C and D) Migrations of sheared cells were evaluated by wound healing assay. (E and F) Invasions of sheared cells were assayed by a transwell device containing Matrigel and then observed by Giemsa dye. Bar = 50 μm. A static treatment was used as a reference to show the relative fold changes of each treatment. Data were provided as means ± SEM. Same letter indicates no significant differences according to Fisher's least significant difference (LSD) post hoc test. Values of *p* < 0.01 were considered as significant. EMT, epithelial–mesenchymal transition.

Monitoring the expression of proteins involved in EMT found that epithelial proteins such as E‐cadherin and ZO‐1 were increased, whereas the mesenchymal proteins Snail and Twist 1 were relatively decreased (Figure 4A,B). In the wound healing assay, the static cells were greatly closing up, whereas the cells treated with shear flow were significantly retarded (Figure 4C). A similar malignant potential was also observed in the invasion assay, where SW620 cells from the static treatment were more invasive compared to the cells treated by shear flow for 1 and 2 h (Figure 4D).

### 3.5 Mitochondrial function under shear flow

**FIGURE 5:**
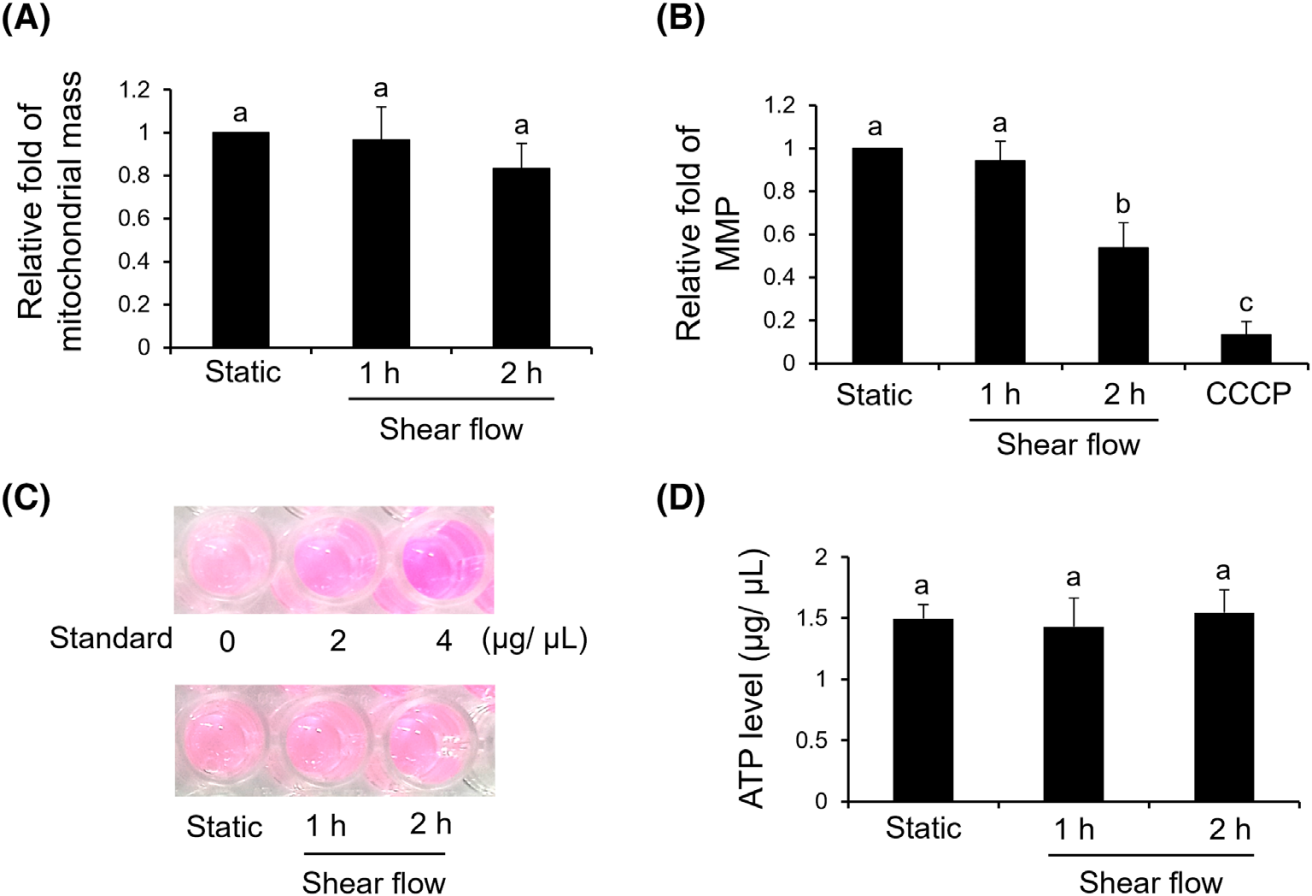
The effect of shear flow on mitochondrial function. (A) After shear flow, the mitochondria were purified from 1 × 10^6^ cells and then the protein concentrations were determined by BCA reagent. (B) Sheared cells (1 × 10^5^) and the cells pre‐treated with CCCP were incubated with JC‐1 reagent and then subjected to flow cytometric analysis. (C and D) Sheared cells (2 × 10^5^) were stained by ATP detecting reagent and then compared to the standard and were determined by ELISA plate reader. A static treatment was used as a reference to show the relative fold changes of each treatment. Data were provided as means ± SEM. Same letter indicates no significant differences according to Fisher's least significant difference (LSD) post hoc test. Values of *p* < 0.01 were considered as significant. CCCP, carbonyl cyanide m‐chlorophenyl hydrazone.

Under shear flow for 1 and 2 h, the protein level determined from isolated mitochondria of sheared SW620 cells was not changed (Figure 5A). As for the MMP, SW620 cells sheared for 1 h did not change significantly compared to the control. However, extended shear flow for 2 h showed a significant decline of the MMP (Figure 5B). Investigating the ATP amount revealed that shear flow did not alter the level of ATP (Figure 5C,D).

### 3.6 Shear flow decreased EMT

**FIGURE 6:**
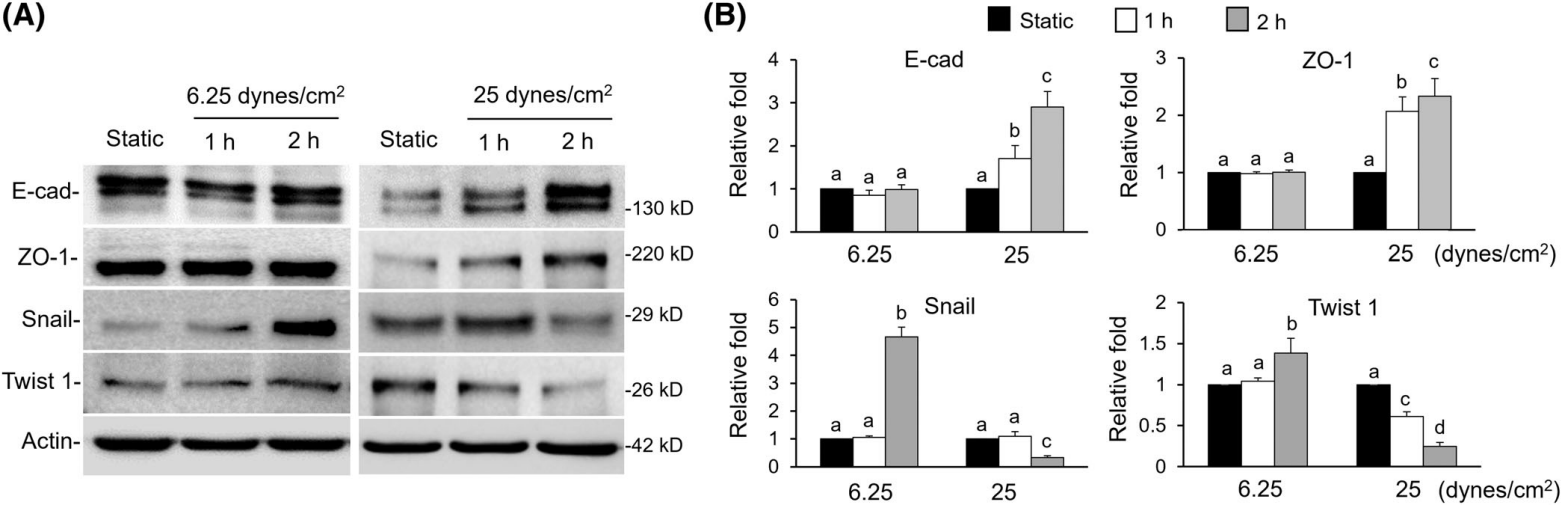
The effect of diverse shear flow on EMT expression. (A and B) Cells were subjected to different strengths of shear flow, namely 6.25 and 25 dynes/cm^2^ for 1 and 2 h. Expressions of E‐cad, ZO‐1, Snail, and Twist 1 were identified by western blotting. A static treatment was used as a reference to show the relative fold changes of each treatment. Data were provided as means ± SEM. Same letter indicates no significant differences according to Fisher's least significant difference (LSD) post hoc test. Values of *p* < 0.01 were considered as significant. EMT, epithelial–mesenchymal transition.

Comparing EMT proteins under diverse shear flow treatments showed that lower shear flow (6.25 dynes/cm^2^) did not change the expressions of the epithelial biomarker proteins E‐cadherin and ZO‐1, whereas they promoted the expressions of the mesenchymal proteins Snail and Twist 1. With an elevated flow speed of 25 dynes/cm^2^, the epithelial proteins were increased, and the mesenchymal proteins were reduced (Figure 6A,B).

## 4 DISCUSSION

Mechanical signals constantly stimulate metastatic CTCs circulating in the bloodstream. Investigating the responses of CTCs on shear flow is, therefore, of potential importance for the improvement of cancer therapy. The applied shear flow device was modified from our previous studies.^24^, ^29^ The cells inside the device encountered at least three types of mechanical forces in addition to shear force, that is, compression and tensile strain. In addition, the cells were also stimulated by rolling and by cells bumping at each other. Therefore, it remains a major challenge to unify the conditions of shear flow and to reduce the influence of irregular flow generated by multidirectional stopcock in the circulation tube. The results from Figures 1 and 2 showed that only apoptosis of SW620 cells was observed, whereas there were no indications of necrosis. This indicated the feasibility of using a rotary pump in performing a shear flow experiment.

In this study, shear flow increased cell size, so the malignant potential of SW620 was thought to be reduced. In general, malignant cancer cells with higher metastatic characteristics showed a smaller cell size due to a deformable and contractile cytoskeleton. Therefore, the cell size of a solid tumor is commonly larger than that of metastatic cancer cells.^30^, ^31^ In addition to enlarged cell size, shear flow also arrests cell cycle at the G1 phase. Investigating the mitochondrial function of sheared SW620, we found that the MMP was significantly decreased whereas the ATP level was not changed. Combining these observations, we expect shear flow shifts to allocate most of the energy equivalents ATP to enlarge the cells and less ATP for cell metastasis. According to published records, the slower dividing cells are always correlated with larger cell size.^32^, ^33^ Similar responses as between cell size and metastasis can be observed for skin and colon cancers for a shift of balance between epithelial and mesenchymal characteristics of cancer cells. At equal energy supply, ameliorated malignancy is correlated with an increase of the epithelial form of cancer cells and a reduction of the mesenchymal form.^34^, ^35^ For the shift of ATP or the balance of EMT in regulating cancer malignancy, the possible implications of membrane proteins such as aquaporin and channels for regulating osmosis and cytoskeleton responses are worth of further study.^36^, ^37^

In the present study, migrating cells of SW620 cells were treated by shear flow for 1 and 2 h and then cultured in culture plates for 24 h. The confluently growing cells were then subjected to a wound‐healing assay for another 24 h. As for the invasion assay, the sheared SW620 cells were seeded on the surface of a Matrigel and then incubated for 24 h to evaluate the invasion rate. Both treatments revealed that even mechanical shear flow treatments for only 1 or 2 h decreased cell migration and invasion for at least 24 h. Therefore, coupled with a newly designed device as discussed previously,^26^ the patients just need dialysis treatment for 2 h and the circulating metastatic SW620 cells would be reduced or even eliminated. This provides a reasonable potential for a combination of chemo‐, immuno‐, and mechano‐therapy in improving the treatment of CTCs.

## 5 CONCLUSION

The present study identified that the malignancy of circulating CRC cancer cells SW620 can be reduced by shear stress. Whether our findings on SW620 cells can be applied to CTCs of other cancer cells warrants further investigation. The present study provides a valuable indication for combining different therapeutic strategies, such as a combination of chemo‐, immuno‐, and mechano‐therapy, in a holistic cancer therapy of the future.

## CONFLICT OF INTEREST STATEMENT

The authors declare no conflict of interest.
